# Supplementary material for: Hybrid Models and Biological Model Reduction with PyDSTool
Source: PLoS Comput Biol. 2012 Aug 9;8(8):e1002628. doi: 10.1371/journal.pcbi.1002628 (PMC3415397; doi:10.1371/journal.pcbi.1002628)
Supplement: Text S4 — Complete source code for the PyDSTool package (version 0.88.120504). Includes API documentation and help files linking to web pages. This file is identical to the current public release on Sourceforge.net. (ZIP) [file pcbi.1002628.s004.zip › PyDSTool/html/PyDSTool.Toolbox.optimizers.line_search.barzilai_borwein_search-pysrc.html]

xml version="1.0" encoding="ascii"?


PyDSTool.Toolbox.optimizers.line\_search.barzilai\_borwein\_search


| Home | Trees | Indices | Help | PyDSTool |
| --- | --- | --- | --- | --- |

Package PyDSTool ::
Package Toolbox ::
Package optimizers ::
Package line\_search ::
Module barzilai\_borwein\_search

[hide private]

[frames] | no frames]

# Source Code for Module PyDSTool.Toolbox.optimizers.line\_search.barzilai\_borwein\_search

```
 1  ##class Function(object): 
 2  ##  def __call__(self, x): 
 3  ##    return (x[0] - 2) ** 2 + (2 * x[1] + 4) ** 2 
 4  ## 
 5  ##  def gradient(self, x): 
 6  ##    return numpy.array((2 * (x[0] - 2), 4 * (2 * x[1] + 4))) 
 7   
 8  import sys 
 9  sys.path.append('/home/dmitrey/scikits/openopt/scikits/openopt/solvers/optimizers') 
10   
11       
12  from line_search import CubicInterpolationSearch 
13  from numpy import * 
14  from numpy.linalg import norm 
15   


16 -def BarzilaiBorwein(function, x0, df = None, maxIter = 1000):


17      x0 = asfarray(x0) 
18      lineSearch = CubicInterpolationSearch(min_step_size = 0.0001) 
19      g0 = function.gradient(x0) 
20       
21      #if norm(g0) <= self.gradtol: return x0 
22      if norm(g0) <= 1e-6: return x0 
23       
24      state0 = {'direction' : g0} 
25   
26      x1 = lineSearch(origin = x0, state = state0, function = function) 
27      print x1 
28      s0 = x1 - x0 
29       
30      y0 = state['gradient'] - g0 
31       
32      #if norm(state['gradient']) <= self.gradtol: return newX 
33      if norm(state['gradient']) <= 1e-6: return x1 
34       
35      xk = x1 
36      sk_ = s0 
37      yk_ = y0 
38      gk_ = state['gradient'] 
39       
40      for k in xrange(maxIter): 
41          alpha_k = dot(sk_, sk_) / dot(sk_,yk_) 
42          sk_ = -alpha_k * gk_ 
43          xk += sk_ 
44          gk_prev = gk_.copy() 
45          gk_ = function.gradient(xk) 
46          yk_ = gk_ - gk_prev 
47          #if norm(gk_) < self.gradtol: return xk 
48          if norm(gk_) <= 1e-6:  
49              print 'k=', k 
50              return xk 
51      return xk

52   
53  if __name__ == '__main__': 


54 -    class Function:


55 -        def __call__(self, x): return ((x-arange(x.size))**2).sum()


56 -        def gradient(self, x): return 2*(x-arange(x.size))

57       
58      x0 = sin(arange(1000)) 
59      fun = Function() 
60      x_opt = BarzilaiBorwein(fun, x0) 
61      print x_opt 
62      print fun(x_opt) 
63
```

  


| Home | Trees | Indices | Help | PyDSTool |
| --- | --- | --- | --- | --- |

|  |  |
| --- | --- |
| Generated by Epydoc 3.0.1 on Fri May 4 15:24:12 2012 | http://epydoc.sourceforge.net |
